# Assessment of ATP Metabolism to Adenosine by Ecto-Nucleotidases Carried by Tumor-Derived Small Extracellular Vesicles

**DOI:** 10.21203/rs.3.rs-3876953/v1

**Published:** 2024-01-24

**Authors:** Chang-Sook Hong, Elizabeth V. Menshikova, Theresa L. Whiteside, Edwin K. Jackson

**Affiliations:** University of Pittsburgh School of Medicine; University of Pittsburgh School of Medicine; University of Pittsburgh School of Medicine; University of Pittsburgh School of Medicine

**Keywords:** tumor-derived small extracellular vesicles, N6-etheno-ATP, N6-etheno-ADP, N6-etheno-AMP, N6-etheno-adenosine, high pressure liquid chromatography

## Abstract

**Background:**

Immunosuppression is a hallmark of cancer progression. Tumor-derived small extracellular vesicles (sEV), also known as TEX, produce adenosine (ADO) and can mediate tumor-induced immunosuppression.

**Methods:**

Here, the ATP pathway of ADO production (ATP◊ADP◊AMP◊ADO) by ecto-nucleotidases carried in sEV was evaluated by a novel method using N^6^-etheno-ATP (eATP) and N^6^-etheno-AMP (eAMP) as substrates. The “downstream” N^6^-etheno-purines (ePurines) were measured by high performance liquid chromatography with fluorescence detection (HPLC-FL).

**Results:**

Human melanoma cell-derived TEX (MTEX) metabolized eATP to N^6^-etheno-ADP (eADP), eAMP and N^6^-etheno-Adenosine (eADO) more robustly than control keratinocyte cell-derived sEV (CEX); due to accelerated conversion of eATP to eADP and eADP to eAMP MTEX and CEX similarly metabolized eAMP to eADO. Blocking of the ATP pathway with the selective CD39 inhibitor ARL67156 or pan ecto-nucleotidase inhibitor POM-1 normalized the ATP pathway but neither inhibitor completely abolished it. In contrast, inhibition of CD73 by PSB12379 or AMPCP abolished eADO formation in both MTEX and CEX, suggesting that targeting CD73 is the preferred approach to eliminating ADO produced by sEV.

**Conclusions:**

The noninvasive, sensitive, and specific assay assessing ePurine metabolism ± ecto-nucleotidase inhibitors in TEX enables the personalized identification of the ecto-nucleotidase primarily involved in ADO production in patients with cancer. The assay could guide precision medicine by determining which purine is the preferred target for inhibitory therapeutic interventions.

## INTRODUCTION

Suppression of adaptive and innate immune responses is a recognized hallmark of cancer progression[1]. In this regard, tumor-derived small extracellular vesicles (sEV), otherwise known as tumor-derived exosomes or TEX, play a key role in tumor-induced suppression of immune effector cells in cancer and in promotion of tumor growth[2, 3]. TEX are small virus-size sEV derived from the endocytic compartment of tumor cells[4]. They are present in all body fluids of patients with cancer, circulate freely and serve as a communication network between malignant and nonmalignant cells[5, 6]. As sEV are secreted by normal and malignant cells, TEX represent a subset of total circulating sEV that can be identified by a unique surface protein profile that mimics that of parent cancer cells[7, 8]. TEX exert direct immunoregulatory effects on immune cells, re-program functions of normal tissue cells located in the tumor-microenvironment (TME) and by autocrine or paracrine mechanisms support tumor growth[9, 10]. TEX are potentially useful as biomarkers of tumor progression and of immune responses or as potential predictors of response to cancer therapies[11, 12].

Numerous in vitro and in vivo studies in animal models demonstrate autocrine effects of TEX on tumor growth, tumor resistance to chemotherapies and establishment of metastases[13–15]. Via juxtacrine or paracrine signaling in the TME, TEX are known to alter functions of mesenchymal stem cells, fibroblasts, endothelial cells and immunocytes[16, 17]. TEX-mediated changes in recipient cells are the result of receptor-ligand interactions on the cell surface and/or up-take by recipient cells of signaling proteins or micro-RNAs (miRs) carried by TEX[18, 19]. As TEX circulate freely delivering pro-tumor and anti-immune response signals to a broad variety of cells, they represent a major mechanism of tumor immune escape.

Among various immunosuppressive molecular pathways utilized by TEX, the adenosinergic pathway is emerging as a major contributor to TEX-induced suppression of immune cells and a promoter of tumor growth and metastasis[20, 21]. TEX-derived adenosine (ADO) inhibits T-cell activation and proliferation through A_2A_Rs[22]. Our studies show that of 20 different purines measured, ADO is the most abundant purine in TEX[23]. Thus, intra-vesicular ADO carried by TEX, which are present in high numbers (e.g.,10^12^/mL) in cancer patients’ plasma, would be efficiently delivered to near or distantly located recipient cells in the TME. Therefore, TEX may be a major source of immunosuppressive ADO, as well as ADO mediating pro-tumor activities in patients with cancer.

Although tumor cells produce and release ADO into extracellular space, ADO is rapidly (within seconds) metabolized and quickly depleted. On the other hand, ADO preformed in tumor cells, packaged, and delivered to recipient target cells by TEX is protected and functionally active[22, 23]. Also, ecto-nucleotidases involved in ADO production, CD39 and CD73, are present on the surface of TEX and are able to convert ATP to ADO [21]. Thus, in addition to intra-vesicular ADO, TEX are equipped with CD39 which converts ATP to ADP and ADP to AMP and with CD73 which metabolizes AMP to ADO[22]. TEX appear to sustain immunosuppressive levels of ADO near target cells by utilizing efficient ecto-enzymatic machinery to manufacture ADO in the extracellular compartment close to cell surface receptors for ADO.

Since TEX are equipped to generate ADO in the extracellular compartment and thus effectively contribute to tumor-induced immune suppression, the ability to measure adenosinergic activity of TEX in patients with cancer might serve as a useful guide to therapy selection and prognostic considerations. Our preliminary data suggest that levels of TEX-mediated adenosinergic activity differ among cancer patients[23]. Thus, the development of a rapid, sensitive, and specific assay that could guide precision medicine for each patient using TEX is a rational objective. We describe here a novel approach to assessing activity of the ATP pathway (ATP ◊ ADP ◊ AMP ◊ ADO) mediated by ecto-nucleotidases residing on the TEX surface and how to selectively block the ecto-nucleotidases mediating extracellular ADO production in every cancer patient.

## MATERIALS and METHODS

Cell lines. Human melanoma cell line Mel524 and human keratinocyte cell line HaCaT were obtained from the ADCC and used for preparation of melanoma cell-derived sEV (MTEX) and control keratinocyte cell-derived sEV (CEX). The cell lines were grown at 37°C in an atmosphere of 5% CO_2_ in air. Cultures were routinely tested and found to be mycoplasma free. Cells were cultured in RPMI-1640 medium, 1 *%* (v/v) penicillin/streptomycin and 10% (v/v) heat-inactivated fetal bovine serum (FBS, ThermoFisher Scientific, Waltham, MA) previously depleted of extracellular vesicles by ultracentrifugation at 100,000×g for 3h. Cells were cultured in 150 cm^2^ cell culture flasks containing 25 ml of the culture medium. Each flask was seeded with 4×10^6^ cells and following 72h of incubation, supernatants were collected, while the cells were harvested using 2 mL of TrypLE Express (Gibco, Grand Island, NY) and washed in serum-containing medium. For subsequent passages, cells were re-seeded in new flasks using the cell numbers described above Supernatants were collected for isolation of TEX and CEX.

Collection of cell line supernatants for sEV isolation. Cell culture supernatants were combined, and a 50mL aliquot of cell culture supernatant was centrifuged at room temperature for 10min at 2,000×g to sediment cells and cell fragments. Supernatants were transferred to new tubes for centrifugation at 10,000×g at 4°C for 30min. Supernatants were collected and filtrated using a 50mL syringe and a 0.22μm bacterial filter. Afterwards, aliquots of supernatants were concentrated to 1 mL by using Vivacell 100 concentrators (Sartorius Corporation, Bohemia, NY) at 2,000×g[24].

sEV isolation by size exclusion chromatography (SEC). sEV isolation by SEC was previously described and is routinely used in our lab[25]. An aliquot (1 ml) of concentrated supernatant was loaded on a 10 cm-long Sepharose 2B column and was eluted with phosphate-buffered saline (PBS). Individual 1 ml fractions were collected. Fraction #4 containing the bulk of non-aggregated morphologically intact sEV was harvested, concentrated using 100,000 MWCO Vivaspin 500 centrifugal 450 concentrators (Sartorius Corporation) and evaluated for protein, vesicle size and number, molecular content and sEV functions[25].

**Figure 1:**
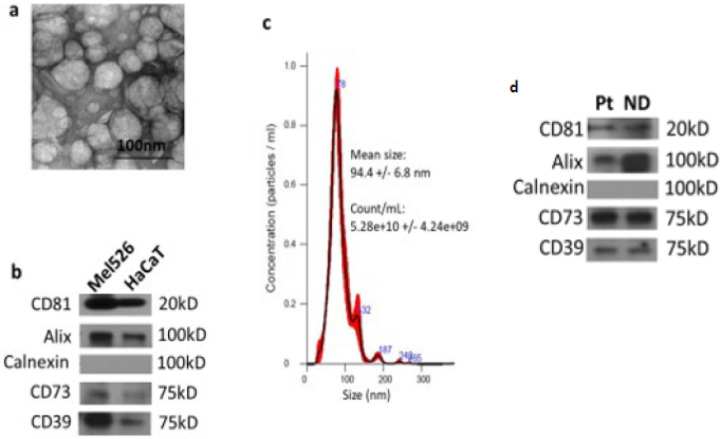
Characterization of sEV. The concentration, size and endocytic origin of sEV in SEC fraction #4 isolated from cell supernatants or human peripheral blood were evaluated. In (**a**) Transmission electron microscopy (TAM) of sEV shows vesicular morphology. The bar = 100nm. In (**b**) Western blots of sEV (**MTEX**) derived from supernatant of a melanoma cell line (Mel526) and of sEV (**CEX**) derived from the supernatant of non-malignant HaCaT cell line. Note the presence of CD39 and CD73, an endosomal marker ALIX, CD81 tetraspanin, and the absence of Calnexin. In (**c**) results of the nanoparticle tracking analysis using NanoSight. The vesicle size ranged from 30–150nm. In (**d**) Western blots of TEX isolated from supernatants of MDA-MB-231(wt) and MDA-MB-231(KO) for CD73. In (**e**) Western blots of sEVs isolated from plasma of a patient with cancer and a healthy donor to illustrate that cell supernatant and plasma derived sEV isolated by the same SEC method have the same protein profiles.

Transmission electron microscopy (TEM). TEM of sEV was performed at the Center for Biologic Imaging, the University of Pittsburgh as previously described[25]. Freshly isolated sEV were placed on copper grids coated with 0.125% Formvar in chloroform and stained with 1% (v/v) uranyl acetate in ddH2O. A JEM 1011 microscope was used for sEV visualization. TEM showed particle morphology consistent with sEVs (see Fig. 1A).

Western blot analysis. To concentrate isolated sEV, 0.5 mL 100K Amicon Ultra centrifugal filters (EMD Millipore, Burlington, MA) were used for centrifugation at 4,000×g. Vesicle aliquots were lysed with Laemmli sample buffer (Bio-Rad Laboratories, Hercules, CA) and separated using 4–15% SDS/PAGE gels. Each lane was loaded with 10μg of fraction #4 protein. After transfer from gels to the polyvinylidene fluoride (PVDF) membranes, proteins were detected using antibodies specific for CD81 (ThermoFisher, MA5–13548), ALIX (ThermoFisher, MA5–32773), calnexin (Cell Signalling, #2433), CD39 (Santa Cruz #33558) and CD73 (Abcam #81720). Immunodetection by blotting showed the protein profile consistent with sEVs (see Fig. 1B).

### NanoSight measurements.

The concentration and size distribution of sEV were measured by nanoparticle tracking analysis (NTA) using NanoSight 300 (Malvern, UK). The vesicles were diluted in ddH_2_O and then the video image was captured at a camera level of 14. The captured videos were analyzed using NTA software, maintaining the screen gain and the detection threshold at 1 and 5, respectively. To determine mean particle size/concentration in each sample, five consecutive measurements were obtained and averaged. NanoSight measurements yielded particles sizes consistent with sEV (see Fig. 1 c).

Protein concentration. Protein concentrations of sEV were determined by using a BCA protein assay (Pierce Biotechnology, Rockford, IL) according to the manufacturer’s instructions.

Functional activity. The ability of isolated sEV (10μg protein) to induce apoptosis of Jurkat T cells during a 6-hour co-incubation was measured by flow cytometry using FITC Annexin-V (ANXV) Apoptosis Detection Kit (BD Biosciences, #55647, Jose, CA) in a Cytoflex flow cytometer (Beckman, Indianapolis, IN) as previously described[26]. Flow cytometry indicated that isolated sEV were functionally active (data not shown).

Preparation of sEV for analysis. Total sEV were concentrated using Amicon ultra-filter (100,000 MWCO) and both MTEX and CEX were prepared in PBS for HPLC-FL analysis at a protein concentration of 100μg/mL. Mean estimated particle concentrations were similar in preparations of MTEX (4.89 × 10^10^/mL) versus CEX (4.26 × 10^10^/mL).

Assessment of N ^6^ -etheno-ATP and N ^6^ -etheno-AMP metabolism by sEV. Cell line-derived sEV were incubated at 37 C in 60μl of PBS with N^6^-etheno-ATP (eATP) or N^6^ -etheno-AMP (eAMP) and without or with enzyme inhibitors. Matched protein amounts (6μg), rather than matched particle numbers, were employed because protein amounts can be measured with greater accuracy than particle counts. Nonetheless, both methods of normalization would provide similar results since at equivalent protein amounts, particle numbers were similar in samples from MTEX versus CEX. High concentrations of eATP and eAMP were employed (100μmol/L) and the incubation periods (3h for eATP; 20min for eAMP) were selected in preliminary experiments to prevent substrate depletion. After incubation, samples were rapidly heat inactivated at 95 C for 90sec to denature ecto-enzymes, centrifuged at 13,000rpm at 4 C and diluted 10-fold before analysis of N^6^ -etheno-Purines (ePurines, BioLog Life Science Institute, Hayward, CA) including N^6^ -etheno-ADP (eADP), eAMP and N^6^ -etheno-Adenosine (eADO). ePurines were quantified using high pressure liquid chromatography with fluorescence detection (HPLC-FL) as recently described by us in detail[27].

We previously determined and reported the sensitivity (detection limit, 1 pmol injected on column), precision (coefficient of variation, < 2%) and accuracy (excellent match between assay values versus known concentrations of standards) of this assay system[27]. Specificity was confirmed by demonstrating baseline separation of all chromatographic peaks generated from samples of a mixture of ePurines and from samples of medium conditioned by four different cell lines incubated with eATP[27]. Moreover, we confirmed that: 1) ePurines are metabolized by ecto-nucleotides with an efficiency similar to their corresponding natural substrates; 2) there is no “off-target” (non-nucleotidase-mediated) metabolism of ePurines; and 3) the metabolism of ePurines is restricted to the membrane surface, i.e., is not due to intracellular nucleotidases[27].

Selectivity assessment of ecto-nucleotidase inhibitors. Many different ecto-nucleotidase inhibitors are available for pharmacological testing of the role of specific ecto-nucleotidases in the metabolism of the extracellular ATP pathway (ATP → ADP → AMP → ADO). However, the selectivity of these inhibitors is uncertain. Therefore, before choosing a given inhibitor to probe the role of a specific ecto-nucleotidase, we tested a panel of commonly used ecto-nucleotidase inhibitors for their selectivity. In this regard, human recombinant CD39 (ENTPD-1), CD203a (ENPP-1), ENTPD-2, ENTPD-3, CD73 and TNAP (R&D Systems, Minneapolis, MN) were incubated with substrate (1 μmol/L) at 30 C for 30 min (with the exception of TNAP which was incubated for 10 min with 50 μmol/L), and the product was measured by HPLC-FL. For all ecto-nucleotidases except CD73, the substrate was eATP; for CD73 the substrate was eAMP With the exception of TNAP the amount of each enzyme was titrated to provide complete conversion of substrate to product within the incubation time in the absence of any inhibitors. For TNAP the amount of enzyme was titrated to minimize the loss of substrate over 10 min because many TNAP inhibitors are non-competitive, and such inhibitors have little effect at low substrate levels. The results of these preliminary studies are summarized in Table 1. The commercial sources of each inhibitor are listed in Table 1.

Statistical analysis. Statistical analysis was conducted using NCSS 2019 Statistical Software (NCSS, LLC. Kaysville, Utah). Data were analyzed with either a Student’s t-test, a 2- factor analysis of variance (2F-ANOVA) or a repeated measures 2-factor analysis of variance (repeated measures 2F-ANOVA) as appropriate. *P*< 0.05 was the criteria for significance. Values are means and SEMs.

## RESULTS

### Characterization of sEV isolated from cell line supernatants.

Figure 1 presents results of sEV characterization performed with MTEX and CEX. The results show that the EV morphology (Fig. 1a), endocytic origin (Fig. 1 b) and size (Fig. 1C) of the vesicles we isolated by ultrafiltration and SEC from supernatants of the two cell lines are consistent with the sEV category. Additionally, western blots showed that these vesicles were positive for CD39 and CD73 (Fig. 1 b). Also, functional assays demonstrated that sEV induced apoptosis of activated T cells in co-incubation assays (data not shown). Vesicles obtained from supernatants of MTEX and CEX EVs (Fig. 1b) and from peripheral blood of a patient with melanoma and a healthy donor (Fig. 1 d) had the same characteristics.

### eATP metabolism by MTEX and CEX.

**Figure 2:**
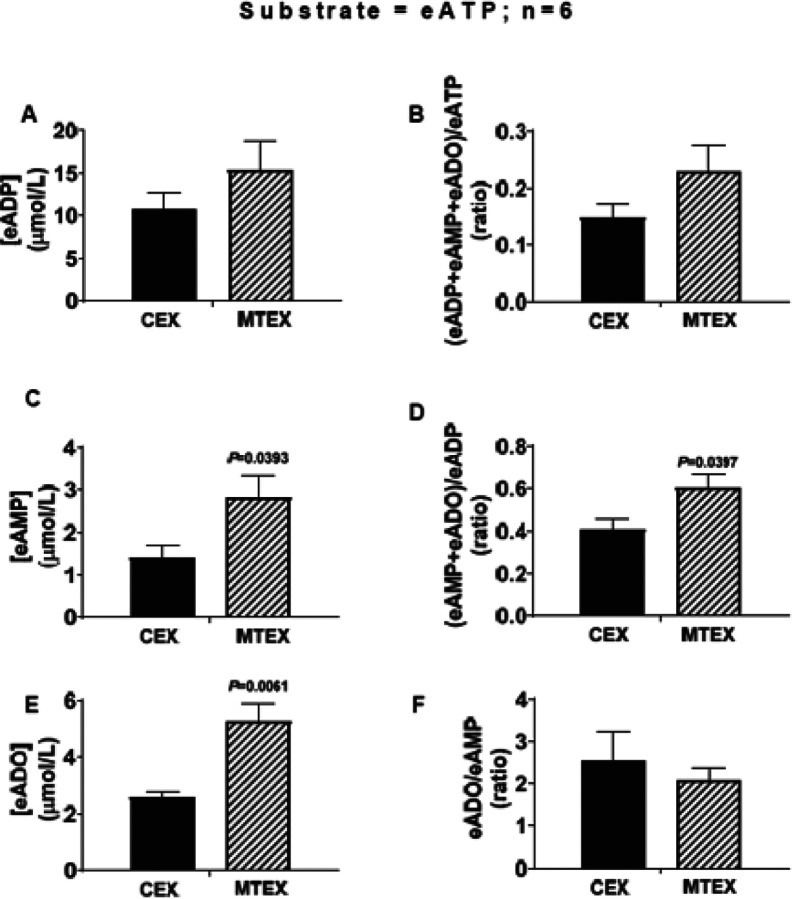
Metabolism of N^6^-etheno-ATP (eATP) to N^6^-etheno-ADP (eADP), N^6^-etheno-AMP (eAMP) and N^6^-etheno- adenosine (eADO) by melanoma cell-derived sEV (MTEX) or control sEV derived from non-malignant keratinocytes (CEX). CEX and MTEX (6μg protein each) were incubated for 3h with eATP (100 μmol/L), and levels of eADP eAMP and eADO were determined. Compared to CEX, eADP concentrations (**A**) tended to be higher in MTEX, while eAMP (**C**) and eADO (**E**) levels were significantly higher in MTEX. The ratio of the sum of “downstream” metabolites levels divided by the immediate “upstream” substrate level also tended to be higher for the substrate eATP (**B**) and was significantly higher for the substrate eADP (**D**),yet was similar for the substrate eAMP (F). The results are consistent with increased metabolism of eATP to eAMP in MTEX versus CEX, which drives increased production of eADO in MTEX. This increased conversion of eATP to eAMP rather than increased metabolism of eAMP to eADO, accounts for the higher production of eADO from eATP in MTEX. Values represent means and SEMs. P-values are from unpaired Student’s t-tests.

MTEX and CEX (6ug protein each) were incubated for 3h with 100μmol/L of eATP; then concentrations of “downstream” ePurines including eADP eAMP and eADO were measured (Figures 2a, 2c and 2e respectively). Notably, the concentrations of eAMP and eADO were higher in MTEX than in CEX (P=0.0393 and P=0.0061, respectively). eADP level tended to be higher in MTEX than in CEX; however, this difference was not statistically significant. We also calculated the ratios of downstream metabolites to upstream substrates as an index of rate of metabolism of a given ePurine. Importantly, the (eAMP+eADO)/eADP ratio was significantly greater (P=0.0397) in MTEX compared with CEX (Figure 2e), and the (eADP+eAMP+eADO)/eATP ratio also tended to be greater in MTEX (Figure 2b). However, eADO/eAMP ratio was similar in MTEX and CEX (Figure 2f). These findings support the conclusion that MTEX metabolize eATP more rapidly to eADP and metabolize eADP more rapidly to eAMP than do CEX. This results in little/no change in steady state eADP levels between MTEX and CEX, yet it increases levels of eAMP in MTEX compared to CEX. The data suggest that increased levels of eADO in MTEX are due to higher eAMP levels driving eADO production.

### Inhibition of CD73 with PSB12379 or AMPCP blocks metabolism of eATP to eADO in MTEX and CEX.

To test the role of CD73 in the metabolism of eATP to eADO, we used α,β-methyleneadenosine 5’-diphosphate (AMPCP); a commonly used CD73 inhibitor. However, our preliminary testing showed that a concentration of AMPCP that suppressed CD73 by 83% also inhibited CD203a (ENPP-1) by 87% (Table 1). By contrast, a concentration of PSB12379, an alternative CD73 inhibitor that blocked CD73 by 98%, had no detectable effect on a panel of ecto-nucleotidases (Table 1). Although CD203a is not known to metabolize AMP to ADO, here we used both AMPCP and PSB12379 to block CD73.

**Figure 3:**
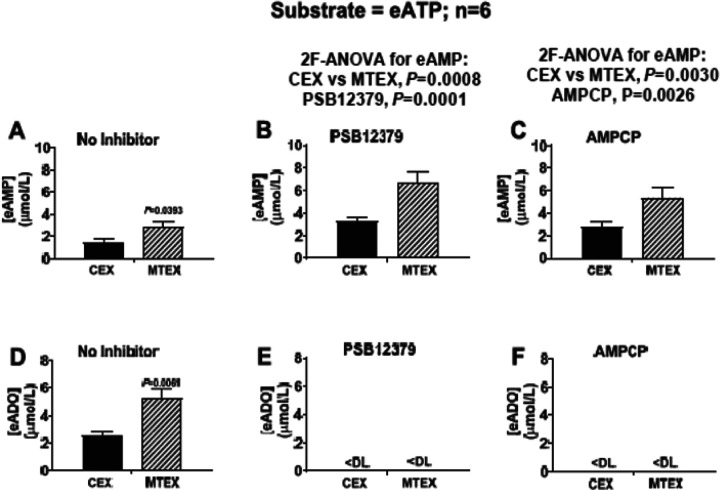
Metabolism eATP to eAMP and eADO in MTEX and CEX in the absence and presence of CD73 inhibition. CEX and MTEX (6ĝ protein each) were incubated for 3h with eATP (100^mol/L) in the absence (No Inhibitor) or presence of a CD73 inhibitor (either PSB12379 or AMPCP), and levels of eAMP and eADO were determined. For the eAMP results (**A, B** and **C**), the No Inhibitor versus the PSB12379 groups and the No Inhibitor versus the AMPCP groups were analyzed by 2-factor analysis of variance (2F-ANOVA). This analysis indicated that eAMP levels were greater in MTEX than in CEX in the absence (**A**) or presence of either PSB12379 (**B**; P=0.0008) or AMPCP (**C**; P=0.0030), a finding consistent with more rapid metabolism of eATP to eAMP in MTEX. Also, in CEX and MTEX, both PSB12379 (**B**; P=0.0001) and AMPCP (**C**; P=0.0026) had elevated levels of eAMP compared to N0 Inhibitor controls, suggesting accumulation of eAMP due to inhibition of its metabolism by CD73. In the absence of CD73 inhibition, eADO levels were higher in MTEX than in CEX (**D**; P=0.0061, Student’s t-test). In the presence of either PSB12379 (**E**) or AMPCP (**F**), eADO was undetectable (DL= lower detection limit of assay), indicating a critical role for CD73 present on the vesicle surface for production of ADO from ATP Values represent means and SEMs.

Consistent with findings reported in Fig. 2, regardless of treatment with PSB12379 (40 ^mol/L) or AMPCP (400 μmol/L), 3hr incubation of vesicles with eATP (100 μmol/L) resulted in a greater increase of eAMP levels in MTEX than in CEX (Figs. 3a–3c; P= 0.0008 for effect of MTEX versus CEX with No Inhibitor versus PSB12379 groups and P= 0.0030 for effect of MTEX versus CEX with No Inhibitor versus AMPCP groups). Also, in both MTEX and CEX incubated with eATP PSB12379 and AMPCP increased eAMP levels (Figs. 3a–3c; P= 0.0001 for effect of PSB12379 versus No Inhibitor; P= 0.0026 for effect of AMPCP versus No Inhibitor). In the No Inhibitor groups, eADO levels were elevated in MTEX compared to CEX (Fig. 3d; P= 0.0061) yet were below detection limit in MTEX and CEX treated with either PSB12379 (Fig. 3e) or AMPCP (Fig. 3f). These findings confirm that MTEX metabolize eATP more rapidly to eAMP and eADO than CEX and demonstrate that in both MTEX and CEX inhibition of CD73 abolishes the conversion of eAMP to eADO resulting in higher levels of eAMP and undetectable levels of eADO.

**Figure 4:**
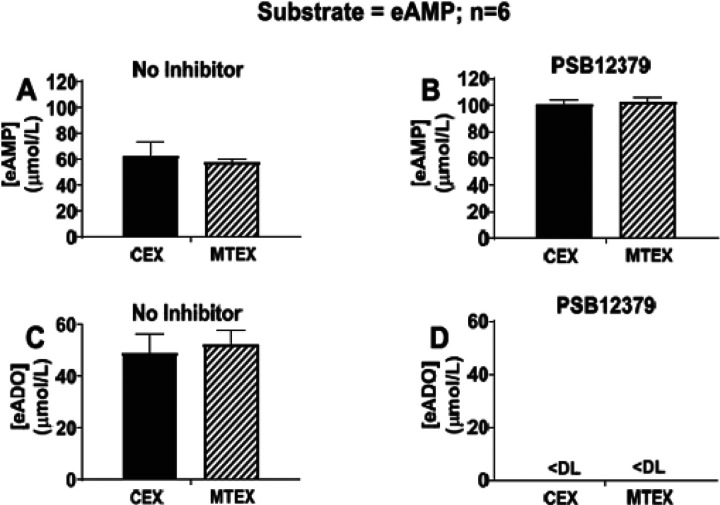
Metabolism eAMP to eADO in MTEX and CEX in the absence and presence of CD73 inhibition. CEX and MTEX (6μjg protein each) were incubated with eAMP (100μmol/L) in the absence (No Inhibitor) or presence of a CD73 inhibitor (PSB12379) for 20 min, and levels of eAMP and eADO were determined. In the absence of PSB12379, approximately half of the eAMP substrate (**A**) was converted to eADO in CEX and MTEX (**C**) during the 20min incubation period. In the presence of PSB12379, all of the eAMP substrate was recovered as eAMP in CEX and MTEX (**B**) and no eADO (**D**) was detected (DL indicates lower detection limit of assay). These results indicate that the metabolism of AMP to ADO is equivalent in CEX and MTEX and emphasizes a critical role of CD73 on the vesicle surface in ADO production from AMP Values represent means and SEMs.

Inhibition of CD73 with PSB12379 blocks metabolism of eAMP to eADO in MTEX and CEX. First, MTEX and CEX were incubated for 20min with 100μmol/L of eAMP in the absence and presence of PSB12379 (40 μmol/L); then eAMP and eADO were measured. In the absence of PSB12379, approximately half of the added eAMP was converted to eADO during the 20min incubation in both MTX and CEX (Figs. 4a and 4c). By contrast, in both MTEX and CEX treated with PSB12379, the added eAMP was recovered as intact, unused eAMP (Fig. 4b) and levels of eADO were below the assay detection limit (Fig. 4d). These results confirm that CD73 mediates the enzymatic conversion of eAMP to eADO in MTEX and CEX and demonstrate that CD73 activity levels are similar in MTEX and CEX.

### Inhibition of tissue nonspecific alkaline phosphatase (TNAP) with L-pbromotetramisole (L-p-BT) does not affect the metabolism of eATP to eADP or eAMP in either MTEX or CEX.

Our results are consistent with the conclusion that CD73 mediates the metabolism of eAMP to eADO in MTEX and CEX. However, the ecto-nucleotidase(s) responsible for converting eATP to eADP and eAMP in MTEX and CEX is (are) unknown. Here, we incubated MTEX and CEX with 100 μmol/L of eATP in the absence and presence of the TNAP inhibitor L-p-bromotetramisole (L-p-BT; 400 μmol/L); then eADP and eAMP were measured. L-p-BT is a commonly used TNAP inhibitor, and our preliminary testing showed that a concentration of L-p-BT that inhibited TNAP activity by 87% had little or no effect on other ecto-nucleotidases (Table 1).

**Figure 5:**
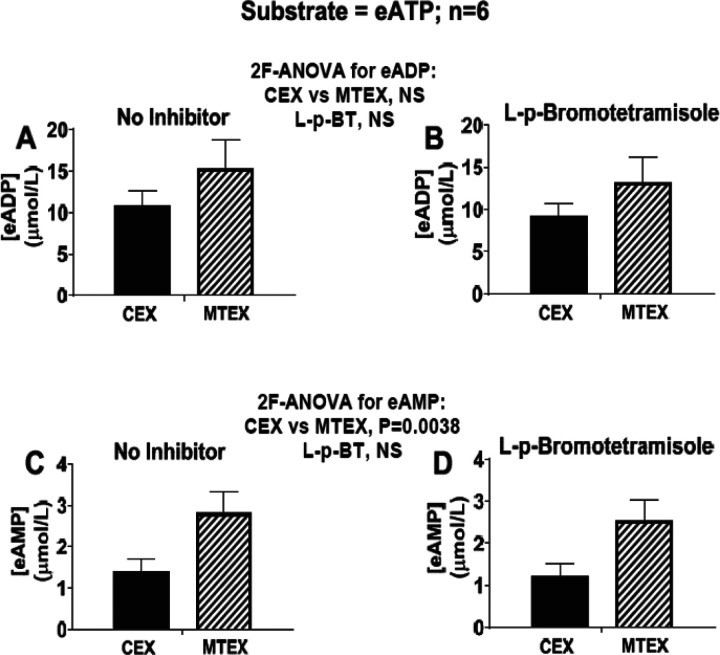
Metabolism of eATP to eADP and eAMP in MTEX and CEX in the absence and presence of inhibition of tissue non-specific alkaline phosphatase (TNAP). CEX and MTEX (6μg protein each) were incubated for 3h with eATP (100μmol/L) in the absence (No Inhibitor) or presence of a TNAP inhibitor (L-p-bromotetramisole; L-p-BT), and levels of eADP (**A** and **B**) and eAMP (**C** and **D**) were determined. Results for the No Inhibitor versus the L-p-bromotetramisole groups were analyzed by 2-factor analysis of variance (2F-ANOVA). This analysis indicated that the production of eADP or eAMP from eATP was not affected by L-p-bromotetramisole. Since CD73 exclusively mediates the metabolism of eAMP in the vesicles (see Figures 3 and 4) and since TNAP is not involved in the conversion of ATP to ADP or AMP TNAP appears not to participate in the ATP pathway in either CEX or MTEX. NS= no significant difference. Values represent means and SEMs.

L-p-BT did not affect the levels of either eADP (Figs. 5a and 5b) or eAMP (Figs. 5c and 5d). As observed earlier, eADP levels tended to be greater in MTEX than in CEX, and eAMP levels were significantly (P=0038) greater in MTEX versus CEX. These results confirm that MTEX metabolize eATP more rapidly than do CEX; however, there appears to be little, if any, role for TNAP in either MTEX or CEX.

### CD39 (ENTPD-1) blockade with ARL67156 inhibits the metabolism of eATP to eADP and eAMP in MTEX and CEX.

ARL67156 is a commonly used CD39 inhibitor. Our preliminary screening experiments indicate, however, that while somewhat selective for CD39, ARL67156 induces considerable blockade of CD203a at concentrations that inhibit CD39 (Table 1) Therefore, we evaluated, using ARL67156 (400 μmol/L) in MTEX and CEX, the role of CD39 to metabolize eATP in the presence of AMPCP (400 μmol/L; blocks CD203a) to remove any influence of CD203a.

**Figure 6:**
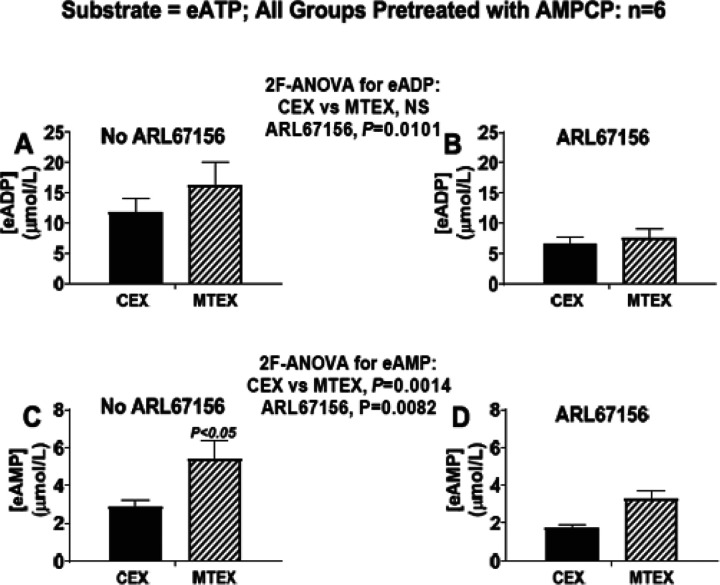
Metabolism of eATP to eADP and eAMP in MTEX and CEX in the absence and presence of selective inhibition of CD39. CEX and MTEX (6μg protein each) were incubated for 3h with eATP (100μmol/L) in the absence (No ARL67156) or presence of a selective CD39 inhibitor (ARL67156), and levels of eADP and eAMP were determined. Since ARL67156 also blocks CD203a (see Table 1), all groups were pretreated with AMPCP which also blocks CD203a (see Table 1), thus isolating the effects of ARL67156 to CD39 inhibition. The results for eADP (**A** and **B**) and eAMP (**C** and **D**) in the No ARL67156 versus the ARL67156 groups were analyzed by 2-factor analysis of variance (2F-ANOVA). This analysis indicated that the production of eADP (P=0.0101) or eAMP (P=0.0014) from eATP was suppressed by ARL67156 in CEX and MTEX and that the excessive metabolism of eATP to eAMP was normalized by ARL67156. However, some eATP was converted to eADP and eAMP in CEX and MTEX despite inhibition of CD39. NS= no significant difference. Values represent means and SEMs.

ARL67156 reduced eADP (Figs. 6a and 6b; P= 0.0101) and eAMP (Fig. 6c and 6d; P= 0.0082) levels in MTEX and CEX and attenuated the excessive eAMP produced in MTEX compared with CEX (Figs. 6c and 6d). These findings support the conclusion that CD39 contributes to the metabolism of eATP to eADP and eAMP in both MTEX and CEX and that excessive CD39 activity accounts for the differential metabolism of eATP to eAMP in MTEX.

CD39 (ENTPD-1) blockade with POM-1 inhibits the metabolism of eATP to eADP and eAMP in MTEX and CEX. POM-1, like ARL67156, is also a commonly used CD39 inhibitor. Our preliminary screening experiments indicate, however, that POM-1 is a pan-ecto-nucleotidase inhibitor (i.e., a non-selective CD39 inhibitor) that induces considerable blockade of not only CD39, but also ENTPD2, ENTPD3, CD203a and TNAP (Table 1). Therefore, to evaluate the combined role of CD39, ENTPD2, ENTPD3 and TNAP we examined the effects of POM-1 (600 μmol/L) on the ability of MTEX and CEX to metabolize eATP in the presence of AMPCP Here, the pretreatment with AMPCP was employed to match the conditions used for assessment of the actions of ARL67156 on eATP metabolism.

**Figure 7:**
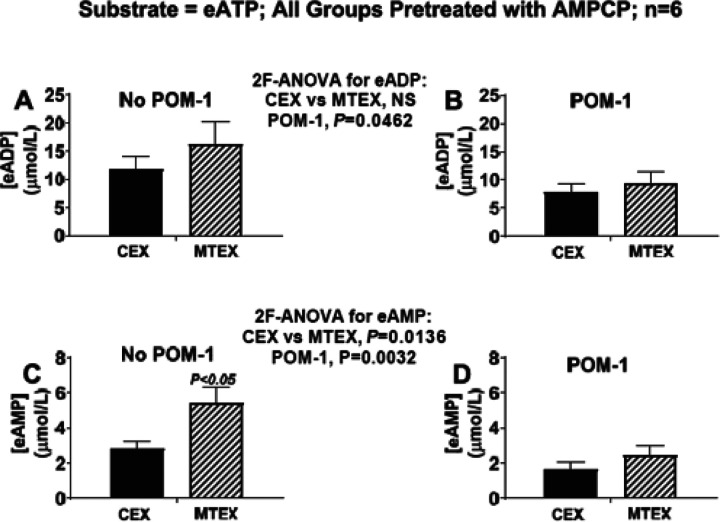
Metabolism of eATP to eADP and eAMP in MTEX and CEX in the absence and presence of pan-ectonucleotidase inhibition. CEX and MTEX (6μg protein each) were incubated for 3h with eATP (100μmol/L) in the absence (No POM-1) or presence of a non-selective pan-ecto-nucleotidase inhibitor (POM-1; blocks CD39, ENTPD2, ENTPD3, CD203a and TNAP see Table 1), and levels of eADP and eAMP were determined. All groups were pretreated with AMPCP so that the results with POM-1 were obtained under the same conditions as in the ARL67156 experiments (see Figure 6). The results for eADP (**A** and **B**) and eAMP (**C** and **D**) for the No POM-1 versus the POM-1 groups were analyzed by 2-factor analysis of variance (2F-ANOVA). This analysis indicated that the production of eADP (P=0.0462) or eAMP (P=0.0032) from eATP was suppressed by POM-1 in CEX and MTEX and that the excessive metabolism of eATP to eAMP was normalized by POM-1. However, some eATP was converted to eADP and eAMP in CEX and MTEX despite inhibition of multiple ecto-nucleotidases. NS = no significant difference. Values represent means and SEMs.

POM-1 reduced eADP (Figs. 7a and 7b; P= 0.0462) and eAMP (Fig. 7c and 7d; P= 0.0032) levels in MTX and CEX and attenuated the excessive eAMP produced in MTEX compared with CEX (Figs. 7c and 7d). The effects of POM-1 were nearly identical to the effects mediated by ARL67156 (compare Fig. 6 with **7**). These findings support the conclusion that CD39 contributes to the metabolism of eATP to eADP and eAMP in MEX and CEX and that excessive CD39, but not ENTPD2, ENTPD3 or TNAP activity, accounts for the differential metabolism of eATP to eAMP in MTEX.

**Figure 8:**
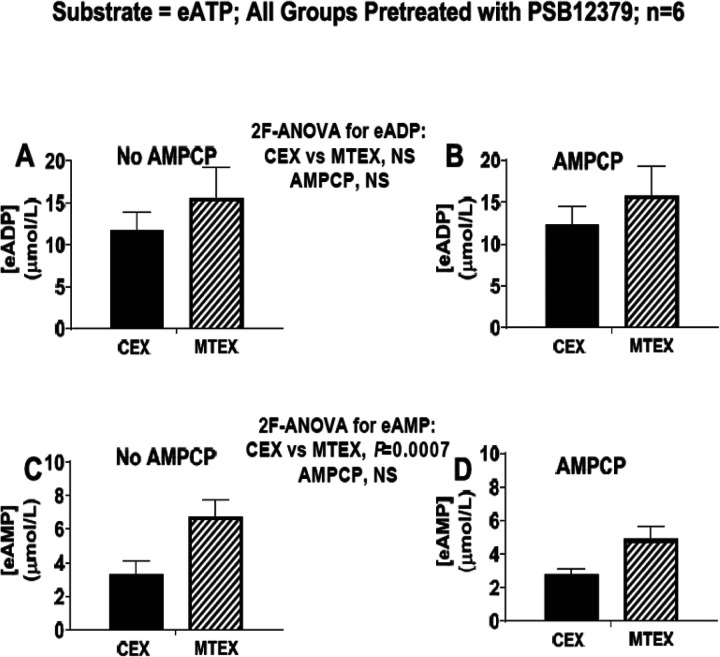
Metabolism of eATP to eADP and eAMP in MTEX and CEX in the absence and presence of CD203a inhibition. CEX and MTEX (6ĝ protein each) were incubated for 3h with eATP (100μmol/L) in the absence (No AMPCP) or presence of a CD203a inhibitor (AMPCP; see Table 1), and levels of eADP and eAMP were determined. Since AMPCP also blocks CD73 (see Table 1) all groups were pretreated with PSB12379 which also blocks CD73 (see Table 1), thus isolating the effects of AMPCP to CD203a inhibition. Results for eADP (**A** and **B**) and eAMP (**C** and **D**) comparing the No AMPCP versus the AMPCP groups were analyzed by 2-factor analysis of variance (2F-ANOVA). This analysis indicated that the production of eADP or eAMP from eATP was not affected by AMPCP in either CEX or MTEX. There appears to be little/no role for CD203a in the metabolism of ATP by the vesicles. NS = no significant difference. Values represent means and SEMs.

CD203a inhibition with AMPCP does not affect the metabolism of eATP to eADP or eAMP in either MEX or CEX. Based on the results described above, it appeared that CD39 is the dominant ecto-nucleotidase in sEV that regulates eATP metabolism. Nonetheless, we considered that CD203a might be involved as well. Accordingly, we evaluated the metabolism of eATP in MTEX and CEX in the absence and presence of AMPCP (400 μmol/L; blocks CD203a). Since AMPCP also inhibits CD73 we conducted these experiments in the presence of PSB12379 (40 μmol/L; blocks CD73) to remove any influence of CD73. AMPCP did not affect the levels of eADP (Figs. 8a and 8b) or eAMP (Figs. 8c and 8d). As observed earlier, eADP levels tended to be greater in MTEX compared with CEX, and eAMP levels were significantly (P= 0.0007) greater in MTEX than in CEX. These results confirm that MTEX metabolize eATP more rapidly than do CEX; however, there appears to be little, if any, role for CD203a in either MTEX or CEX.

## DISCUSSION

The primary goal of this study was to determine whether the method utilizing ePurines and HPLC-FL we previously developed was adequately sensitive to study ATP metabolism to ADO in sEV. These vesicles were previously found to carry ADO in the lumen as well as ecto-nucleotidases on the external surface and to produce ADO [21]. We reported that TEX had augmented ATP metabolism to ADO and, considering the role of ADO in cancer-induced immune suppression, wished to precisely quantitate ADO generated by TEX of different patients with cancer. The rationale for this study was supported by our earlier observations that ePurines can be measured with high sensitivity and specificity using HPLC-FL, because the etheno moiety renders ePurines fluorescent[27], and this approach does not require more expensive technology such as tandem mass spectrometry. The assay we performed utilizes N^6^ -etheno-ATP (eATP) and N6-etheno-AMP (eAMP) as substrates while measuring “downstream” N^6^-etheno-purines (ePurines) using high performance liquid chromatography with fluorescence detection (HPLC-FL). The assay has the capacity to discriminate not only high from low adenosinergic activity, but also, by the use of specific inhibitors, to identify the ecto-nucleotidases involved in orchestrating the metabolism of external ATP in sEV thus indicating which enzyme inhibitors would be optimal for blocking this immunosuppressive mechanism.

There are several advantages to using ePurines, rather than natural purines, to evaluate the importance of ADO produced by sEV. As we described recently, ePurines are metabolized by ecto-nucleotidases with efficiencies similar to that of corresponding natural purines, yet ePurines can be measured with much greater sensitivity and specificity using HPLC-FL because the etheno moiety renders ePurines fluorescent[27]. Also, because ePurines do not significantly cross membranes and are not conveyed into cells by nucleoside/nucleotide transporters[27], the metabolism of ePurines by EVs would only reflect metabolism by the ecto-nucleotidases with the proper orientation on the sEV surface to produce ADO in the extracellular compartment. Since ADO receptors exist on surfaces of various cells, it is the pool of extracellular ADO that is most important for inducing immunosuppression. Also, ePurines are not metabolized by “off-target” pathways, e.g., deamination, which allows for more precise estimates of the metabolism rate specifically via the extracellular ATP ◊ ADP ◊ AMP ◊ ADO pathway. Finally, the application of inhibitors of ecto-nucleotidases carried by sEV can facilitate future clinical utility of the assay by identifying which ecto-nucleotidase(s) should be targeted for inhibition.

The experiments we performed comparing MTEX and CEX yielded several interesting results. First, in all sEV, the main ecto-nucleotidases participating in the “extracellular ATP to ADO pathway” are CD39 and CD73; TNAP ENTPD2, ENTPD3 and CD203a play little if any role. The use of pharmacological inhibitors which selectively blocked individual enzymes in the pathway confirmed the involvement of CD39 and CD73 in the ATP to ADO pathway in both MTEX and CEX.

The finding that the “Extracellular ATP to ADO Pathway” is significantly enhanced in MTEX relative to CEX represents another important observation, which agrees with previous reports that tumor cells producing sEV which carry intraluminal ADO and actively produce ADO are the major source of immunosuppressive ADO in cancer plasma [22, 28]. Furthermore, enhanced enzymatic activity of CD39 converting ATP to ADP to AMP was responsible for the higher activity of the entire pathway in MTEX compared to CEX. This finding also fits well with higher ATP utilization by cancer cells and cancer-derived sEV [21]. Upon the addition of eATP to sEV, eAMP and eADO, but not eADP levels were increased approximately 2-fold more in MTEX versus CEX, suggesting that MTEX metabolize eATP more rapidly to eADP and metabolize eADP more rapidly to eAMP than do CEX. Nevertheless, metabolism of eAMP to eADO remains similar in MTEX and CEX. This is because increased production and increased utilization of eADP by CD39 normalizes eADP levels and leaves them relatively constant, while increasing eAMP levels and ADO production, which is what we observed. Since CD39 metabolizes both ATP and ADP the enhanced metabolism of eATP in MTEX is consistent with similar eADP levels in MTEX and CEX, although eAMP and eADO levels were twice as high in MTEX compared to CEX.

Selective inhibition of CD39 with ARL67156 or pan-ecto-nucleotidase inhibition with POM-1, which inhibits multiple ecto-nucleotidases, including CD39 and at least ENTPD2, ENTPD3, TNAP and CD203a, similarly normalized the conversion of eATP to eAMP This suggests that CD39, not the other ecto-nucleotidases inhibited by POM-1, mediates the accelerated utilization of eATP in MTEX. Also, inhibition of CD203a did not affect the metabolism of eATP which further rules out the involvement of this ecto-nucleotidase in the metabolism of eATP in sEV. Importantly, although both ARL67156 and POM-1 normalized the metabolism of eATP in MTEX to that observed in CEX, neither inhibitor abolished eATP metabolism in either MTEX or CEX. This suggests that other, yet to be identified ecto-nucleotidases on the surface of sEV might also contribute to metabolism of eATP but are not responsible for the accelerated metabolism of eATP by MTEX. Our experiments rule out involvement of TNAP in this step since L-p-BT did not alter the metabolism of eATP in either MTEX or CEX. In aggregate, these experiments showed that the enhanced ATP to ADO pathway in MTEX is due to higher activity of CD39 and results in increased production of eAMP (a substrate for eADO) in MTEX.

We next considered the role of CD73 in the enhanced ATP to ADO pathway in MTEX and found that CD73 activity was not responsible for increased levels of eADO production in MTEX versus CEX. This conclusion is supported by two lines of reasoning. First, incubation with eATP yielded similar eADO-to-eAMP ratios in MTEX and CEX. Second, incubation with eAMP demonstrated equal metabolism of eAMP by MTEX versus CEX. Notably, inhibition of CD73 with either PSB12379 or AMPCP reduced eADO levels in both MTEX and CEX to below the detection limit of our assay. So, although CD73 does not account for the accelerated metabolism of eATP to eADO in MTEX, CD73 in both MTEX and CEX exclusively mediates the conversion of eAMP to eADO. These findings suggest that regardless of the upstream source of AMR inhibition of CD73 abolishes ADO production by sEV in both MTEX and CEX.

In conclusion, measurements of ePurine metabolism in sEV, with or without ecto-nucleotidase inhibitors, using HPLC-FL could guide precision medicine by identifying patients with ADO-generating ecto-nucleotidases in TEX and by determining how best to block ADO production by TEX. Although the relative importance of ecto-nucleotidases in TEX will likely vary among patients and may depend on the type of cancer, stage of cancer and exposure to therapeutic agents, the present results suggest that CD73, rather than CD39, inhibition might be a preferred mode of therapy in cancer patients.

## Figures and Tables

**Table 1:** Effects of inhibitors on recombinant ecto-nucleotidases.

INHIBITOR	Ecto-Nucleotidase					
	CD39 (ENTPD1)	ENTPD2	ENTPD3	CD203a (ENPP-1)	CD73	TNAP
**ARL67156 (200 μmol/L)**	93% inhibition of eAMP formation	No Inhibition	39% inhibition of eAMP formation	74% inhibition of eAMP formation	3% inhibition of eADO formation	37% inhibition of eATP metabolism
**AMPCP (200 μmol/L)**	No inhibition	No Inhibition	No Inhibition	87% inhibition of eAMP formation	83% inhibition of eADO formation	25% inhibition of eATP metabolism
**DIDS (220 μmol/L)**	76% inhibition of eAMP formation	54% inhibition of eAMP formation	98% inhibition of eAMP formation	73% inhibition of eAMP formation	2% inhibition of eADO formation.	96% inhibition of eATP metabolism
**L-p-BT (200 μmol/L)**	No inhibition	No Inhibition	No Inhibition	14% inhibition of eAMP formation.	No inhibition	87% inhibition of eATP metabolism
**POM-1 (300 μmol/L)**	94% inhibition of eAMP formation	90% inhibition of eAMP formation	96% inhibition of eAMP formation	87% inhibition of eAMP formation	No inhibition	81% inhibition of eATP metabolism
**PPADS (120 μmol/L)**	95% inhibition of eAMP formation	27% inhibition of eAMP formation	96% inhibition of eAMP formation	54% inhibition of eAMP formation	No inhibition	86% inhibition of eATP metabolism
**PSBO69 (200 μmol/L)**	74% inhibition of eAMP formation	92% inhibition of eAMP formation	68% inhibition of eAMP formation	97% inhibition of eAMP formation	No inhibition	38% inhibition of eATP metabolism
**PSB12379 (20 μmol/L)**	No inhibition	No Inhibition	No Inhibition	No inhibition	98% inhibition of eADO formation	No Inhibition
**Suramin (720 μmol/L)**	88% inhibition of eAMP formation	93% inhibition of eAMP formation	98% inhibition of eAMP formation	96% inhibition of eAMP formation	No inhibition	61% inhibition of eATP metabolism

CD39, CD203a, ENTPD-2, and ENTPD-3: substrate, eATP (1 μM); temperature, 30°C; time, 30 min.

CD73: substrate, eAMP (1 μM); temperature, 30°C; time, 30 min.

TNAP: substrate, eATP (50 μM); temperature, 30°C; time, 10 min.

CD39, CD203a, ENTPD-2, ENTPD-3, and CD73: Enzyme amount was titrated to provide complete conversion of substrate to product in 30 min at 30°C (CD39, 20 ng; CD203a, 80 ng; ENTPD-2, 40 ng; CD73, 40 ng; ENTPD3, 20 ng).

TNAP: Enzyme amount was 5 ng and was titrated to minimize loss of substrate over 10 min due to uncompetitive inhibition in which uncompetitive inhibitor has little effect at low substrate levels.

Inhibitor concentration was selected to suppress by >70% CD39 (ARL67123, PPADs, Suramin, DIDS, PSB069), TNAP (L-p-BT) or CD73 (AMPCP PSB12379).

**ARL67156**: Millipore Sigma, Burlington, MA

**AMPCP**: α,β-Methyleneadenosine 5’-diphosphate; Millipore Sigma, Burlington, MA

**DIDS**: 4,4’-Diisothiocyanato-2,2’-stilbenedisulfonic acid disodium salt; Tocris Biosciences, Minneapolis,MN

**L-p-BT**: L-p-bromotetramisole; Santa Cruz Biotechnology, Dallas, TX

**POM-1**: Tocris Biosciences, Minneapolis, MN

**PPADS**: Pyridoxalphosphate-6-azophenyl-2’,4’-disulfonic acid; Tocris Biosciences, Minneapolis, MN

**PSB069**: Tocris Biosciences, Minneapolis, MN

**PSB12379**: Tocris Biosciences, Minneapolis, MN Suramin: Tocris Biosciences, Minneapolis, MN

## Data Availability

The data are available from the authors upon personal request.
